# Reply

**DOI:** 10.1111/jch.14410

**Published:** 2021-12-11

**Authors:** Jiunn‐Tyng Yeh, Tzu‐Jung Chiu, Hao‐Min Cheng

**Affiliations:** ^1^ Department of Medicine National Yang Ming Chiao Tung University College of Medicine Taipei Taiwan; ^2^ Department of Medical Education Taipei Veterans General Hospital Taipei Taiwan; ^3^ Center for Evidence‐based Medicine Taipei Veterans General Hospital Taipei Taiwan; ^4^ Institute of Public Health National Yang Ming Chiao Tung University College of Medicine Taipei Taiwan; ^5^ Institute of Health and Welfare Policy National Yang Ming Chiao Tung University College of Medicine Taipei Taiwan

We appreciate the questions raised by Dr. Giménez‐Miranda and associates. To begin with, we would like to address the inherent limitation of the observational study, which is the type of research included into our systematic review and meta‐analysis.^1^ Due to the inability of randomization and, for some, retrospective design, the conventional analysis of the observational studies (eg, calculating odds ratio or hazard ratio) can only infer correlation instead of causation. Techniques such as causal inference have been developed to determine causality from observational studies, yet in our meta‐analysis, the effect estimates we extracted and synthesized are associative in nature.^2^

For the hypothesis proposed by Dr. Giménez‐Miranda and associates that dysautonomia resulting from certain subtypes of dementia causes blood pressure variability (BPV), we agree that it is biologically and clinically plausible.^3^, ^4^ However, our results may not be interpreted based on this mechanism due to the following reasons. First, most of the studies included in our analysis measured the BPV on a visit‐to‐visit basis, which is proposed to reflect the adherence and compliance to antihypertensive agents or measurement errors and aging.^5^ BPV subtypes that are more correlated with dysautonomia, such as orthostatic hypertension or nocturnal blood pressure fall, were excluded during our evidence synthesis process. Second, an important criterion inferring causation is the temporal sequence of association.^6^ The observational studies included in our analysis measured BPV in advance, then the incidence of dementia or cognitive decline were subsequently evaluated. Certainly, there could be patients with insidious dementia and dysautonomia, which were not detected during the period of blood pressure measurements. To further prove the dysautonomia hypotheses, the temporal sequence of the study should be inversed, that is, research should estimate the change of BPV for patient diagnosed with dementia and compare with those without.

As mentioned in the *Discussion* of our paper, interpretation to the results of our analysis should be made with caution due to the limited number of studies, high heterogeneity, and the absence of a dose‐response relationship. Hence, it is possible that when conducting subgroup analysis based on the etiologies of the dementia, the lack of statistical power can explain the insignificant results. The question on the relationship between specific subtypes of dementia and BPV requires more studies, such as observational studies conducted with a causal‐inference model, or RCTs investigating the cognitive outcome among patients with different BPV targets.

## CONFLICTS OF INTEREST

The authors have no competing interests.
